# Sustainable Development Goals: Challenges and the Role of the International Society of Nephrology in Improving Global Kidney Health

**DOI:** 10.34067/KID.0000000000000237

**Published:** 2023-10-26

**Authors:** Sabine Karam, Michelle M.Y. Wong, Vivekanand Jha

**Affiliations:** 1 Division of Nephrology and Hypertension, University of Minnesota, Minneapolis, Minnesota; 2 Division of Nephrology, Department of Medicine, University of British Columbia, Vancouver, British Columbia, Canada; 3 George Institute for Global Health, UNSW, New Delhi, India; 4 School of Public Health, Imperial College, London, United Kingdom; 5 Prasanna School of Public Health, Manipal Academy of Medical Education, Manipal, India

**Keywords:** CKD

## Abstract

The United Nations 2030 agenda for sustainable development includes 17 sustainable development goals (SDGs) that represent a universal call to end poverty and protect the planet, and are intended to guide government and private sector policies for international cooperation and optimal mobilization of resources. At the core of their achievement is reducing mortality by improving the global burden of noncommunicable diseases (NCDs), the leading causes of death and disability worldwide. CKD is the only NCD with a consistently rising age-adjusted mortality rate and is rising steadily up the list of the causes of lives lost globally. Kidney disease is strongly affected by social determinants of health, with a strong interplay between CKD incidence and progression and other NCDs and SDGs. Tackling the shared CKD and NCD risk factors will help with progress toward the SDGs and *vice versa*. Challenges to global kidney health include both preexisting socioeconomic factors and natural and human-induced disasters, many of which are intended to be addressed through actions proposed in the sustainable development agenda. Opportunities to address these challenges include public health policies focused on integrated kidney care, kidney disease surveillance, building strategic partnerships, building workforce capacity, harnessing technology and virtual platforms, advocacy/public awareness campaigns, translational and implementation research, and environmentally sustainable kidney care.

## Introduction

In 2015, the United Nation General Assembly adopted the 2030 Agenda for Sustainable Development^1^ to secure a more sustainable future.^1^ The 17 sustainable development goals (SDGs) representing a universal call to action to end poverty, protect the planet, and ensure prosperity for all, are intended to guide government policies, mobilize resources, foster international cooperation, and engage stakeholders from all sectors of society, including businesses, civil society organizations, and individuals. Since their adoption, the SDGs have become a framework for measuring progress and promoting sustainable development at all levels.

Noncommunicable diseases (NCD) are the leading causes of death and disability globally and at the forefront of the quest to achieve SDGs. The sole health-related SDG 3 aims at “reduction by one-third premature mortality from NCDs through prevention and treatment” and achieving “universal health coverage (UHC), including financial risk protection, access to quality essential health care services, and access to safe, effective quality and affordable essential medicines and vaccines.”^1^

The global NCD health agenda has focused on cardiovascular disease (CVD), stroke, cancer, diabetes mellitus (DM), and chronic lung disease, while kidney diseases remained neglected.^2^ In addition, progress on kidney diseases is often looked at through the prism of other NCDs such as DM, hypertension (HTN), and CVD rather than considered an epidemiological entity on its own. One possible reason is that CKD, as currently defined and classified, is a term coined relatively recently with a little more than 20 years of existence, while other NCDs have already benefited from advocacy efforts for many decades.^3^ It is nonetheless important to point out that World Health Organization (WHO) states, in item 19 of the United Nation Political Declaration on NCDs,^4^ that “renal, oral and eye diseases … share common risk factors and can benefit from common responses to NCDs.” Health threats due to kidney disease (Box 1) are on the rise.^8^ Indeed, as is the case with other NCDs, kidney disease is strongly affected by social determinants of health, *e.g.*, sex, race/ethnicity, religion, education, place of residence, economic, environmental, and living conditions.^9^ Inequities are especially prevalent in the practice of nephrology compared with other specialties because dialysis and transplant are costly, resource-intensive interventions.^10^ Finally, the relative weights of the drivers of kidney diseases differ according to the socioeconomic settings. Whereas DM and HTN are in general the major drivers of CKD, other causes of kidney disease play a significant role in countries with low sociodemographic index. Moreover, low and middle sociodemographic index countries account for the highest global age-standardized rates of CKD disability-adjusted life years.^11^ Accelerating progress in global kidney health and reaching milestones in the development of all SDGs and in advancing NCD care go together, and appropriate attention must be accorded to kidney disease, the only NCD with rising age-standardized mortality. This review will highlight the linkages, some challenges, and opportunities to achieving SDGs in relation to global kidney health.

**Box 1 Health threats and burden of kidney disease.^5–7^**

- More than 850 million people have kidney disease worldwide.
- 35.8 million annual disability-adjusted life years are attributable to CKD.
- CKD is the only noncommunicable disease with an increasing age-adjusted mortality rate.
- CKD constitutes the third fastest growing cause of death.
- CKD is expected to become the fifth leading cause of years of lives lost worldwide by 2040.
- Thirteen million patients develop AKI every year, and 1.7 million die of it every year.
- One hundred eighty eight million experience catastrophic health care expenditure annually due to kidney disease.
- About 2.7–3 million people are receiving kidney replacement therapy worldwide.
- 93% of the global dialysis population is in high-income/upper-middle-income countries.
- Three to seven million with kidney failure die without kidney replacement therapy every year.

## Interplay between CKD, Other NCDs, and Other SDGs

The global kidney disease incidence is affected by access to primary care for control of risk factors and therefore is more likely to increase in low-resource settings where such access is limited.^12^ In turn, CKD is an important risk factor for NCDs, most notably CVD, and for death.^13^ Therefore, progress on other NCDs has a knock-on effect on kidney disease burden and *vice versa*. Because SDGs and the reduction of NCDs, and by extension kidney diseases, are interconnected and share several common objectives, tackling one of them would automatically be beneficial to the others (Table 1).

**Table 1:** United Nations Sustainable Development Goals and relevance to kidney health (Adapted from ref. 17)

SDG		Relevance to Kidney Health
1	No poverty	• Improved nutrition, personal safety, health care should improve kidney disease prevention, identification, management • Reduced catastrophic health expenditure
2	Zero hunger	• Improved maternal nutrition and reductions in low birth weight and preterm birth which are risk factors for CKD • Improved nutrition can reduce obesity-related risks of CKD, diabetes, HTN
3	Good health and well-being	• Improved prevention, identification, and treatment of kidney disease • Public health programmes to promote healthy lifestyles and vaccinations could reduce AKI and CKD
4	Quality education	• Improved awareness and knowledge of kidney health • Reduced use of nephrotoxic remedies
5	Gender equality	• Reduced adolescent pregnancies, and increased pregnancy spacing should reduce incidence of low birth weight, prematurity, pregnancy-related complications that are risk factors for CKD
6	Clean water and sanitation	• Reductions in waterborne diseases and diarrheal illnesses that are risk factors for AKI and CKD
**7**	Affordable and clean energy	• Broaden use of mobile health in kidney disease prevention and treatment for patients, and education for community workers and health care providers • Improvements in electronic data sharing, and developing registries
8	Decent work and economic growth	• Improvements in access to health care, dignity, and wealth could improve prevention, early identification and treatment of CKD • Improved retention of health care workers • Facilitates task-shifting
9	Industry, innovation, and infrastructure	• Support innovations to improve affordability and sustainability of access for prevention, identification and treatment for CKD and AKI
10	Reduced inequalities	• Improved equity and geographical access to prevention, identification, and intervention for all forms of kidney diseases and all types of kidney care • Improved access to new and/or expensive therapies
11	Sustainable cities and communities	• Improved level of preparedness in mass disasters for patients with CKD, AKI, kidney failure • Urban planning to reduce food deserts and increase physical activity can reduce obesity-related and diabetes-related kidney disease
12	Responsible consumption and production	• Sustainable, environmentally friendly local production of dialysis supplies could reduce costs, and support local economy by creating jobs • Reductions in need for dialysis can reduce carbon footprint
13	Climate action	• Global warming has been associated with CKD of unknown etiology that may be related to dehydration and toxin exposure • Adverse impact of climate change on transmission of pathogens causing infectious diseases and poverty that increase risk of kidney disease
14	Life below water	• Reduction in marine pollution can reduce risk of pollution-related CKD
15	Life on land	• Reduction in leaching of toxins from industrial waste into ground water can reduce risk of pollution-related CKD
16	Peace, justice, and strong institutions	• Reduction in armed conflict could reduce AKI due to crush injuries/major trauma, improve food security, and reduce preterm birth, a risk factor for CKD • Among marginalized populations, improved prevention, identification, and treatment of kidney disease
17	Partnerships for the goals	• Improved global partnerships for health care financing, regulation, and health-related research could improve understanding of kidney disease, reduce inequities in kidney care, and reduce “transplant tourism”

SDG, sustainable development goal; HTN, hypertension.

SDG 2 aims to end hunger, improve nutrition, and promote sustainable agriculture. Poor nutrition, including unhealthy diets and lack of access to nutritious food, is a crucial risk factor for kidney disease development and poor outcome.^14^ Notable is the role of maternal malnutrition on fetal nephron endowment and the development of kidney disease later in life.^15,16^ Promoting sustainable and diverse food systems that prioritize healthy and balanced diets (SDG 12) can also contribute to reducing the development of kidney disease and other NCDs. Better control of CKD can be achieved by promoting diets low in processed foods, sugar, salt, and saturated fats and rich in fruits and vegetables. This would happen through sustainable production and consumption of fresh, locally sourced and nutritious foods, controlling harmful substances (pesticides, pollutants, and additives) and encouraging sustainable lifestyles.

SDG 11 emphasizes creating inclusive, resilient, and safe cities. Urban environments play an increasing role in shaping lifestyles and influencing kidney disease risk factors, such as sedentary behavior, air pollution, and easy and safe commute. Designing cities that prioritize walkability, cycling infrastructure, and clean air would have a favorable effect on the development and/or progression of kidney diseases.

SDG 13, which involves taking urgent action to combat climate change, has far-reaching implications for kidney health. Indeed, global warming is likely contributing to the epidemic of CKD of unknown origin which has been identified primarily in hot, rural regions of the world and has been discussed in greater detail elsewhere.^17,18^ Dehydration can be a risk factor for kidney stones. Increased frequency of wildfires, dust storms, and the production of air pollutants affect air quality, which has been associated with an increased risk of CKD.^19^ Climate change contributes to the occurrence of more frequent and intense natural disasters, which can affect access to essential medications and kidney replacement therapies.

SDG 16, which aims to promote just, peaceful and inclusive societies, plays a vital role in creating an enabling environment and supporting strategies that can indirectly contribute to the control of kidney diseases by ensuring equitable access to health care for people with and at risk of kidney diseases, including prevention, diagnosis, and treatment by improving health care delivery, inclusive decision-making processes, and participatory governance, promoting justice, reducing discrimination, and addressing socioeconomic disparities. SDG 16 also emphasizes the importance of reliable data, indicators, and monitoring mechanisms for effective governance, important for CKD care. Finally, this SDG highlights the significance of peace, stability, and security. Conflict and instability can disrupt kidney care and hinder access to essential services. By fostering peaceful societies, promoting social cohesion, and addressing the root causes of conflict, SDG 16 creates an environment that supports kidney disease control efforts and allows for effective interventions.

SDG 17 recognizes the importance of multistakeholder partnerships in achieving the SDGs through collaboration among governments, international organizations, civil society, and the private sector. Strategic partnerships are vital to resource mobilization, knowledge sharing, and development of innovative solutions to tackle kidney diseases. Until now, approaches to tackling NCDs have taken place in silos, preventing coordination. Given that many NCDs occur together, it makes sense to develop integrated prevention approaches, especially in primary care.

The reduction of kidney diseases contributes to several SDGs indirectly. For instance, by preventing and controlling kidney diseases, countries can promote economic productivity (SDG 8), reduce poverty and inequalities (SDG 1 and SDG 10), promote gender equality (SDG 5), and enhance overall well-being and quality of life (SDG 3).

## Challenges to Kidney Health–Related SDGs

### Related to Socioeconomic Factors

Country-level capacities for the diagnosis and management of kidney diseases vary greatly around the globe. According to the International Society of Nephrology (ISN)–Global Kidney Health Atlas, only half of countries provide public funding for nondialysis CKD care^20^; one-third of low-income countries (LIC) reported ability to measure serum creatinine, and none were able to measure proteinuria in primary care settings.^21^ Moreover, the shortage of all kinds of health care workers is striking in LICs. The management of kidney failure is influenced by the local culture, education, human resources, socioeconomic factors, and political stability. The proportion of people with kidney failure not receiving RRT reaches 96% in LIC.^22^ CKD is responsible for the largest number (188 million) of people suffering catastrophic health care expenditures in lower-middle income countries.^23^ Overall, patients who benefit from RRT comprise approximately 0.15% of the global population but consume a disproportionate percentage of the health care budget^24^ which can generate problems of prioritization and also can be nonsustainable in times of crisis.^25^

### Related to Natural and Man-Made Disasters

Natural or man-made disasters such as pandemics, earthquakes, and wars disrupt health care systems and threaten access to essential kidney care.^25,26^ Coronavirus disease 2019 (COVID-19) revealed the lack of global preparedness to emergencies. In addition to being a communicable disease with a dire effect, COVID-19 profoundly affected NCD care.^27^ Those with preexisting CKD were among the most vulnerable to contracting and dying from the infection. The case fatality rate among infected patients on maintenance dialysis or kidney transplant recipients was 25%.^28^ Armed conflicts lead to the destruction of health care infrastructure and to insecure and chaotic environments as seen during wars in Syria, Yemen, Soudan, Ethiopia, and Ukraine.^29,30^ Challenges are related to shortage of consumables and qualified medical personnel^31^ and safe travel of patients and staff, among others. Undocumented immigrants constitute another vulnerable population. In the United States, a limited number of states provide state-wide access to regular hemodialysis (HD) leaving most of this population to resort to emergency-only dialysis, with resultant physical symptoms, psychosocial distress, and higher death rates along with significantly higher health care costs.^32,33^

## Opportunities to Improve Global Kidney Health

At the core of the SDG framework is the multisectoral approach to address the economic, social, and environmental determinants of health. Each SDG has the potential to improve kidney health and provision of kidney care (Table 1). The resulting socioeconomic benefits, including improved productivity, lower health care costs, and financial risk protection, make a strong health-economic case for addressing kidney diseases as a part of progress toward SDG goals.

Aligned with the ISN's 10 recommendations for global kidney health,^34^ we discuss opportunities to address the challenges of improving kidney health globally and improving access to kidney care under seven main themes.

### Public Health Policies: Framework for Sustainable, Integrated Kidney Programs

**Figure 1:**
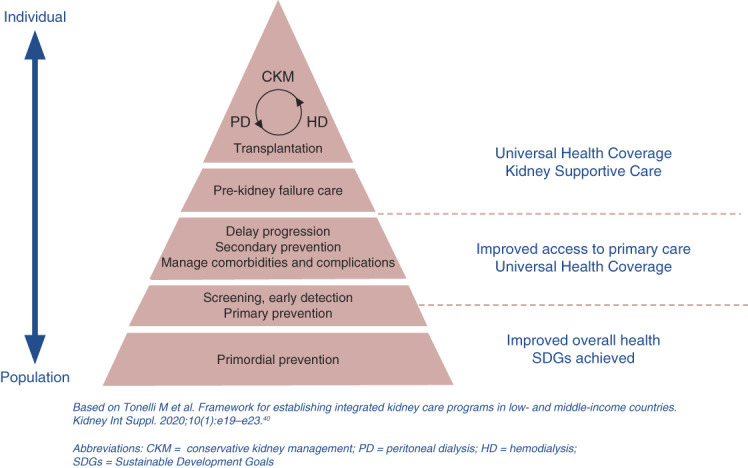
**Components of integrated kidney care.** Source: ISN framework for developing dialysis programs in low-resource settings. ISN, Brussels, Belgium. CKM, conservative kidney management; HD, hemodialysis; ISN, International Society of Nephrology; PD, peritoneal dialysis; SDG, sustainable development goal.

The ISN framework for sustainable, integrated kidney care programs (Figure 1) provides a rational approach that prioritizes cost-effective treatments.^35,36^ Preventive care aimed at early detection and preventing disease progression being the most cost-effective strategy has the highest priority. Preventive care also lends itself to integration with other NCD programs, such as diabetes and CVD. Conservative care programs aimed at managing symptoms of kidney failure should be offered to those who do not have access to or choose not to receive RRT. Criteria for eligibility for RRT should be transparent, equitable, and guided by social values. Among RRT modalities, expanding access to kidney transplant for suitable patients is a priority. Dialysis modality prioritization in LIC/lower-middle income countries (LMIC) should allow for flexibility on the basis of each country's availability and cost evaluations. Peritoneal dialysis (PD) is associated with similar outcomes to HD with lower cost in most countries, but there are regions, such as Africa, in which PD is more expensive than HD because of the high cost of importing PD fluids/supplies.^36,37^ The increased rate of PD uptake during the COVID-19 pandemic^38^ has provided an opportunity to re-evaluate barriers to uptake of home-based modalities.^39^

The WHO UHC Compendium informs a core set of health services and interventions, tailored to population needs to help countries achieve UHC.^40^Strong, person-centered primary health care that emphasizes health promotion and preventive care is the cornerstone of UHC. Point-of-care tools and digital health technologies can facilitate home monitoring by frontline health workers and empower patients with self-management skills^41^ and have been successfully used in several settings, including programs led by community health care workers in India,^42^ and mobile health care teams in rural and remote Indigenous Communities in Canada.^43^

Telemedicine can help facilitate communication between patients, primary health care providers, and nephrologists. Harnessing electronic systems to deliver nephrology care to remote and rural areas can mitigate geographic barriers to care, but more studies are needed to assess their cost-effectiveness and effects on the provider–patient relationship.^44^ Electronic decision-support systems, such as the CKD Pathway^45^ and the Kidney Failure Risk Equation,^46^ can assist health care providers with risk stratification and individualization of CKD management.

There is a great need to improve person-centeredness of kidney care. More than ever, it is important to engage with patients to codesign kidney care programs aligned with their values.^47^ There is a need to expand the circle of stakeholders involved in the generation of evidence to answer questions around the most appropriate programs for society and the evidence-to-policy process arc.^48^ The benefits of a person-centered, culturally safe approach to clinical care, advocacy, research, and policy development include improved health outcomes, improved cost-effectiveness, and improved individual, family, and provider satisfaction.^47,49^

### Surveillance: Establishing Renal Registries

Because the global burden of CKD is underestimated, building renal registries is an important strategy to increase awareness of CKD burden, support health services planning and delivery, and facilitate research. Most high-income countries have renal registries, while many lower-middle income countries do not.^50^ In a global survey of renal registries, over 90% collected adult HD or PD data, and 72% collected adult transplant data. However, few registries collected data on AKI (9%) or nondialysis CKD (22%).^50^ Pediatric dialysis and transplant data were collected by 60%–75% of registries.^50^

The ISN “SHARing Expertise to support the setup of Renal Registries” is supporting development of renal registries globally.^51^ Standardizing data definitions and sharing data can facilitate global advocacy and research efforts.^50^ Other multinational initiatives, including the coronavirus multinational observational registry and the COVID-19 global survey study jointly led by the ISN and the Dialysis Outcomes and Practice Patterns Study, have allowed global sharing of knowledge gained from COVID-19 surveillance in kidney patients.^52^

Improving surveillance of AKI, especially in LIC and LMIC, is one of the aims of the 0by25 initiative to understand prevalence of AKI and to work toward eliminating deaths from AKI worldwide by 2025.^53^

### Building Strategic Partnerships

Because kidney disease often arises from downstream effects of other NCDs, building strategic partnerships with other societies and the WHO can assist with advocating for health policies to curb common risk factors such HTN, obesity, diet, physical inactivity, and smoking.^54^ Aligning the kidney health agenda with other SDGs, such as improved nutrition and maternal health, can help to reduce risk of CKD related to low birth weight and preterm birth.^17^ Second, such partnerships can promote integrated, holistic NCD care models that address the multimorbidity care needs of patients with kidney disease. Third, incorporating kidney health into knowledge translation products of other societies, such as WHO, cardiovascular, diabetes, and HTN societies, improves awareness of kidney disease and its management.

ISN has official relations with the WHO, with a Collaboration Plan to deliver research and advocacy-related projects to address kidney diseases within the context of global, regional, and national NCD strategies.^55^ This collaboration has produced the ISN Framework for Developing Dialysis Programs in Low-Resource Settings.^10^

Public–private partnerships can be instrumental for care provision but require strong oversight and a governance mechanism to ensure value and accountability for public funds and to address potential conflicts of interest. For example, in a public–private partnership model for dialysis care in Georgia, the government has committed to partial funding for dialysis programs, while private dialysis units provide infrastructure, technologies, and training for staff. A regulatory agency was created for oversight and reimbursement.^56^

### Building Capacity to Suit Local Needs

ISN has introduced unique capacity-building programs, including its Fellowship Program which allows persons from low-resource countries to pursue nephrology training abroad; the Interventional Nephrology Training Centers Program; the Sister Centers Program, which pairs developing nephrology centers with established centers to form supportive partnerships; and the Mentorship Program which fosters expertise-sharing relationships between ISN members.^57^ A new Program, the Emerging Leaders Program, brings together early career professionals to work on collective projects to improve the global kidney health agenda worldwide. This program provides cohort members opportunities to interact with experts in public health, ethics, leadership, and advocacy, as well as to participate in ISN working groups. Through this initiative, enduring international networks are created and collaborations facilitated. The 26 members of the first two cohorts are currently engaged in projects evaluating access to essential medicines and sustainable kidney care, as well as implementation projects to improve CKD identification, post-AKI follow-up, and green nephrology.

During the COVID-19 pandemic, increased use of virtual education platforms democratized access to webinars, conferences, and continuing medical education. For example, the World Congress of Nephrology 2021, hosted on a virtual platform, included significantly more attendees from LIC/LMIC countries compared with the previous live World Congress of Nephrology in 2019.^58^ Other virtual capacity-building programs include the GlomCon Fellowship, a glomerular disease fellowship that brings together clinicians, pathologists, and researchers.^59^ This program has been particularly beneficial in regions where there is a lack of local access to glomerular disease experts and educators.

While increasing the number of nephrologists and allied health professionals is a long-term solution to addressing the current gap in the nephrology workforce, a more immediate endeavor should be aimed at cross-competency training, especially in LIC and LMIC. Examples of task-shifting^60^ include educating primary care in management of CKD and AKI in community settings, and upskilling nonphysician health care workers on CKD identification and lifestyle management of CKD. To facilitate this, the ISN-Kidney Disease Improving Global Outcomes CKD Early Identification and Intervention Toolkit^61^ particularly targeted at primary care physicians and allied health professionals, was developed. Future surveys of the global nephrology workforce need to be more inclusive to accurately assess the non-nephrologist workforce, which outnumbers nephrologists several-fold.

### Campaigns to Raise Public Awareness of Kidney Disease

Several initiatives have been established to raise awareness of the global burden of kidney disease among policymakers and the public. World Kidney Day, a joint initiative of the ISN and International Federation of Kidney Foundations, was established in 2006.^62^ With an overall focus on “Kidney health for all,” each year World Kidney Day has a different theme that focuses on various aspects of kidney health.^63^

Advocacy at the community level can be highly effective. Examples include health care providers engaging with the general public through community events and social media, in addition to CKD screening programs run by nongovernmental organizations and locally trained volunteers.^64^

### Translational and Implementation Research

Across the translational research continuum, there are two main gaps that threaten the adoption of novel therapeutic agents into clinical practice: the translational gap between preclinical research and human studies, and the implementation gap from clinical research to real-world implementation in health care settings.

To address the translational gap, the ISN developed a new consensus guidance for optimizing the design and conduct of animal studies for development of new drugs for kidney disease (TRAnslational Nephrology Science FOR new Medications). Their recommendations highlight the importance of animal model standardization, with the goal of helping accelerate drug development in nephrology.

On the other end of the spectrum, addressing global kidney care issues through implementation research is key to tailoring evidence-based solutions to context-specific settings. Implementation research helps to identify barriers associated with delivering and sustaining interventions or policies; and to define interventions at the level of policy, community, organization (*e.g.*, reminder systems and clinical audits), physician (*e.g.*, education through key opinion leaders or other strategies) and/or patient (*e.g.* text messaging; education campaigns through mass media).^60^ These strategies can accelerate adoption of evidence-based practices and policies, which in turn can improve the health of populations and assist with achieving SDGs.^65^

With the addition of renoprotective agents to improve outcomes in patients with CKD, optimizing the implementation of these newer therapies and other foundational, evidence-based therapies should be the focus of future research. Access to disease-modifying medications that can prevent progression to kidney failure is especially important in LIC and LMIC where RRT may not be readily accessible or affordable. This will require ongoing efforts at all levels, from advocating for government health policies to achieve UHC, negotiating with drug manufacturers to reduce pricing for essential medicines,^66^ improving supply/distribution systems, and providing prescriber education. Local champions, national/regional nephrology societies, nongovernmental organizations, and patient organizations have important roles in facilitating implementation of guidelines and health policies.^60^ Support of training and infrastructure to conduct mixed-methods research, pragmatic trials, and learning health system studies, suited to assessing real-world effectiveness and implementation, are also necessary. The ISN-Advancing Clinical Trials is an initiative to improve the capacity of the global nephrology community to participate in clinical trial research.^67^

### Sustainable Kidney Care

Sustainable kidney care to optimize planetary health has emerged as an important theme that relates to virtually all SDGs, most notably SDG 11 (Sustainable cities and communities), 12 (Responsible consumption and production), SDG 13 (Climate Action), SDG 6 (Clean water and sanitation), and SDG 7 (Zero hunger). MacNeill *et al.* outlined a framework for sustainable health systems with three key principles, which were adapted to nephrology, including (*1*) reducing demand for health services by prioritizing health promotion, disease prevention, and chronic disease management; (*2*) matching the supply of health services to demand through resource stewardship and goals of care discussions; and (*3*) reducing emissions from the provision of health services, including green infrastructure/operations, home-based therapies, and virtual care.^68,69^ Under the principles of green nephrology,^70^ environmentally sustainable approaches can be implemented across all forms of kidney care. Plant-based diets promote planetary health through more environmentally sustainable food production and potential kidney health benefits for patients.^71^ While dialysis is very resource-intensive, strategies such as recycling reverse osmosis reject water, using renewable energy sources, and recycling of plastics/cardboard can improve the carbon footprint of dialysis units.^72^

### Conclusion

With its global burden rising, kidney disease has become one of the leading causes of death worldwide. As a NCD, kidney disease is strongly affected by social determinants of health and inequities. All SDGs are interconnected and relevant to achieving optimal kidney health. Challenges to global kidney health are rooted in many factors. Opportunities to address these challenges require a multipronged approach.
